# How well can physicians manage Tuberculosis? A Public-Private sector comparison from Karachi, Pakistan

**DOI:** 10.1186/1472-6963-13-439

**Published:** 2013-10-25

**Authors:** Maliha Naseer, Ali Khawaja, Amin S Pethani, Salik Aleem

**Affiliations:** 1 Division of Environmental Health Sciences, Department of Community Health, Sciences, The Aga Khan University Hospital, Stadium Road, P.O. Box-3500, Karachi (74800), Pakistan; 2 Medical College, The Aga Khan University Hospital, Stadium Road, P.O. Box-3500, Karachi (74800), Pakistan; 3 Department of Community Health Sciences, The Aga Khan University Hospital, Stadium Road, P.O. Box-3500, Karachi (74800), Pakistan; 4 Medical College, Ziauddin Medical University Hospital, Karachi, Pakistan

**Keywords:** Public private mix, TB-DOTS, Pakistan, NTP

## Abstract

**Background:**

Tuberculosis (TB) is endemic in Pakistan which ranks fifth amongst the twenty two countries designated to be highly burdened by TB according to the World Health Organization. However, there is paucity of data regarding the knowledge of diagnosis of TB and its management amongst public and private practitioners. In this study, we endeavor to identify this gap in knowledge regarding the diagnosis and management of TB between public and private doctors and the factors affecting these knowledge scores in urban Pakistan.

**Methods:**

This cross sectional survey was conducted between June and December 2011. Doctors from public hospitals, private hospitals and private clinics scattered in all eighteen towns of Karachi were included in the study. Qualified MBBS doctors working in any specialty were eligible to participate whereas doctors working in both the public and private sectors were excluded from the study. Vignette based clinical scenarios were given to assess the knowledge score regarding the diagnosis and management of TB.

**Results:**

A total of 196 doctors participated in the study. There was a significant difference between private and public physicians in terms of age and years of practice (p-value <0.05). Significant differences in the proportion of knowledge scores were observed between the public and private doctors and National TB Control Program trained and untrained doctors in Karachi. Factors associated with inadequate knowledge scores were being female gender [OR: 2.76 (95% CI: 1.418-5.384)], private employment status [OR: 1.50 (95% CI: 1.258-2.439)], and not trained by NTP [OR: 2.98 (95% CI: 1.286-3.225)] on multivariate logistic regression analysis.

**Conclusion:**

It is concluded that a knowledge gap exists between the public and private doctors in Karachi. Strengthening of currently implemented public private mix model along with improvement in the trainings of public and private practitioners is highly recommended to control TB in Pakistan.

## Background

Tuberculosis (TB) remains a major health problem despite the aggressive preventive and control measures taken in the past few decades [1]. It is responsible for an estimated 8.8 million cases and 1.4 million deaths globally [2]. Eighty-five percent of these TB cases are concentrated in Asia and Africa where there is a lack of education, health care infrastructure, poverty and overcrowding [3].

Pakistan is ranked fifth amongst the twenty two countries designated to be highly burdened by TB and accounts for 63% of the TB burden in the World Health Organization (WHO) Eastern Mediterranean Region [4]. It is also estimated to have the fourth highest prevalence of multidrug-resistant tuberculosis (MDR-TB) globally [5]. With an aim to eliminate TB, National Tuberculosis Control Program (NTP) Pakistan endorsed the WHO initiated Directly Observed Treatment Short Course (DOTS) in 1995 and in 2001, declared TB a national emergency [6].

Since then, a steady progress has been made in TB case detection and treatment success rate but it is still far away from meeting the targets related to Millennium Development Goal (MDG) 6 by 2015 [7].

Pakistan has a mixed type of health care system that comprises public and private formal/informal sectors. In addition, there is an expanding network of 42,700 registered private facilities that are involved in provision of healthcare services; most of these are clinics, chemist shops (69%) and medical stores (27%). The private health delivery network also consists of 550 private hospitals. Furthermore, there are doctors who simultaneously work in the public and private sectors [8,9]. Like many other developing countries, studies have shown that the majority of patients in Pakistan initially attend a private health care provider prior to suspicion of TB [10]. Hence, there is now a growing recognition to enhance public private partnership in controlling TB in Pakistan. Government initiated a public-private mix model in 2003 with the purpose to bring case detection rate up to a target of 70% and treatment success up to 85% through active involvement of care providers from the private sector [11].

Proper TB case management is essential in eliminating TB and health care providers play a crucial role in its diagnosis and management [12]. It not only includes 6–8 months of treatment with anti-tuberculosis drugs but also involves counseling regarding the disease process and adherence to treatment. Failure to do so by care providers, either due to lack of knowledge or motivation, may lead to maltreatment with relapse and spread of drug resistant organisms.

Evidence suggests that private practitioners in developing countries are not equipped with sufficient knowledge to carry out proper case management of a TB patient [13-16].

There is paucity of data regarding the knowledge of diagnosis of TB and its management amongst public and private practitioners and only few studies have been conducted in Pakistan to assess the knowledge of private practitioners [17,18]. There is a need to identify the knowledge gap between public and private doctors and the factors that are associated with good knowledge, attitude and practices of physicians in urban areas of Pakistan. This would be beneficial for policy makers, program managers and care providers in preparing policies and interventions to strengthen the currently implemented public private mix model in the country.

### Objectives

Objectives of the study were as follows:

1. To identify the gap in knowledge regarding the diagnosis and management of TB between public and private doctors in urban Pakistan.

2. To identify the factors affecting the knowledge scores regarding the diagnosis and management of TB among doctors in urban Pakistan.

## Methods

### Study design and study population

A cross sectional survey was conducted between June and December 2011 in public and private health care facilities of Karachi. Karachi is a mega city and the financial hub of Pakistan with an estimated population of over 21 million people [19]. Health care is provided by qualified and unqualified medical professionals (quacks) and according to the Pakistan Medical and Dental Association, the total number of registered MBBS doctors are 53,101 (31,117 males and 21,984 females) in Sindh, Pakistan [20]. In this study, qualified MBBS doctors working in any specialty were eligible to participate and doctors who were working in both the public and private sectors were excluded from the study.

### Sampling technique

#### Selection of health care facility

Private tertiary care hospitals (Ziauddin Hospital, Liaquat National Hospital and National Medical Centre) and government health care facilities (Civil hospital, Lyari General Hospital, Sobhraj Maternity Hospital, Liaquatabad General Hospital and Ojha Institute of Chest Diseases) were selected purposively. In addition, general practitioners working independently in selected Union Councils (UCs) of Kemari, Gulshan-e-Iqbal, Gulberg, Baldia and Korangi towns were also enrolled in the study. These health care facilities (hospitals and private general practitioners) cater for population with great cultural diversity in terms of ethnicity, language and religion.

However, patients belonging to low socioeconomic class are more likely to visit public hospitals and general practitioners’ clinics as compared to patients from middle to higher socioeconomic class who usually prefer private hospitals as a source of treatment [21].

#### Selection of doctors

Doctors working in selected health care facilities were invited to participate through convenient sampling technique. Permission to collect information was sought from the administration of the hospitals. However, hospitals’ administration was not involved in selection and recruitment of the study participants.

### Sample size

Sample size was calculated using Open Epi software version 2.3.1. A previous study from India showed that doctors in the public sector had 2.1 times better knowledge than doctors from the private sector (odds ratio 2.1; P = 0.05) [22]. Taking power to be 80% to detect statistical difference between public and private doctors and 95% confidence interval, sample size was calculated to be 188 qualified medical practitioners (94 in each group). In this study we collected data from 196 physicians; 100 from public hospitals and 96 from the private sector.

### Data collection

Data was collected through a self-administered structured questionnaire. It included two parts; the first part aimed to collect the socio-demographic information and professional characteristics of doctors such as qualification, years of practice, number of patients treated daily, number of TB patients treated/followed in the last one year and whether they have attended the standard NTP training on management of TB. The second part focused on TB and intended to assess the clinical knowledge of the doctors regarding its diagnosis and management. It consisted of five clinical vignettes and 20 multiple choice questions related to the diagnosis, treatment, contact screening and follow up of a patient with TB. A vignette can be designed to assess the knowledge as well as the doctor’s skills in performing the tasks necessary to diagnose and manage a patient. It has been found to be an accurate tool for measuring the quality of physician care in an outpatient setting [23]. The clinical vignettes were adopted from national TB manual for physician published by NTP, Ministry of Health Pakistan. The questionnaire was also reviewed and finalized by the specialist physicians and public health experts working for control of TB.

Data was collected mainly on the working days in isolation by a self-administered questionnaire after informed consent. No incentive was given to study participants. The study was approved by the Ethical review Committee of the Department of Community Health Sciences, The Aga Khan University Karachi, Pakistan.

### Statistical analysis

Data entry and analysis were performed on Epi-Data version 3.1 and Statistical package for social science SPSS (Release 19.0 standard version, copyright © SPSS), respectively. Socio-demographic and professional characteristics of public and private doctors were analyzed using descriptive statistics and compared using chi-square test. Knowledge of TB diagnosis, management and counseling characterized as good, fair and poor were computed and compared between public vs. private doctors and trained vs. untrained by NTP by using chi-square test. To assess the association of socio-demographic and professional characteristics of doctors on knowledge, univariate and multivariate logistic regression analysis were performed using dichotomous knowledge variable [*adequate*: knowledge score >75%, *inadequate*: knowledge score ≤ 75%].

## Results

A total of 128 doctors from public hospitals, 76 from private hospitals and 47 private practitioners working in different localities were approached to participate in the study out of which 100, 58 and 38 gave the consent to participate, respectively.

**Figure 1:**
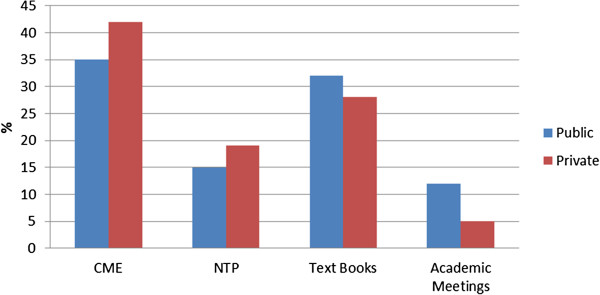
The opinion of participating physicians regarding the sources of knowledge for diagnosis and management of Tuberculosis.

Socio-demographic and professional characteristics of the doctors are presented in Table 1. A significant difference was observed in age and years of practice between both the groups (p-value <0.05). Doctors working in the private sector were younger than those practicing in the public sector. Female representation was relatively low in both groups. Around 50% of the doctors had diploma or fellowship certifications after the basic MBBS degree, out of which most belonged to the public sector. Although almost one third of the doctors were not trained by NTP on DOTS methodology, 50% of them followed the DOTS guidelines for diagnosing and treating patients. Majority (90%) of the study participants had a positive notion on involvement of doctors from the private sector in TB DOTS program. Doctors trained by NTP had significantly higher mean knowledge scores compared to those who were not trained (p-value < 0.001). Overall, Continuing Medical Education (CME) was found to be the main source of knowledge for both the public and private doctors followed by textbooks, NTP trainings and academic meetings (Figure 1).

**Table 1:** Socio-demographic and professional characteristics of public and private physicians in urban Pakistan

**Variable**	**Public**	**Private**	**All**	**p-value**
	**n = 100 (%)**	**n = 96 (%)**	**n = 196 (%)**	
**Age in years**				
20-35	29 (29.0)	46 (48.0)	75 (38.0)	0.038^*^
36-50	46 (46.0)	25 (26.0)	71 (36.0)	
>50	25 (25.0)	25 (26.0)	50 (26.0)	
**Sex**				
Male	56 (56.0)	66 (69.0)	122 (62.0)	0.452
Female	44 (44.0)	30 (31.0)	74 (38.0)	
**Qualification**				
MBBS	46 (46.0)	55 (57.0)	101 (51.5)	0.50
Diploma/Fellow	54 (54.0)	41 (43.0)	95 (48.5)	
**Years of practice**				
≤10 years	37 (37.0)	59 (61.5)	96 (49.0)	0.001^*^
>10 years	63 (63.0)	37 (38.5)	100 (51.0)	
**NTP Training**				
Yes	29 (29.0)	32 (33.0)	61 (31.0)	0.255
No	71 (71.0)	64 (67.0)	135 (69.0)	
**Follow DOTS methodology**				
Yes	47 (47.0)	50 (52.0)	97 (50.0)	
No	36 (36.0)	30 (32.0)	66 (33.0)	0.388
Don’t know	17 (17.0)	16 (16.0)	33 (17.0)	
**Opinion regarding involvement of private physicians in TB Dots program**				
Yes	91 (91.0)	87 (91.0)	178 (91.0)	
No	5 (5.0)	4 (4.0)	9 (4.5)	0.254
Don’t know	4 (4.0)	5 (5.0)	9 (4.5)	

*p-value <0.05 was considered as significant.

Tables 2 and 3 show differences in the proportion of knowledge scores between public and private doctors and NTP trained versus untrained doctors. Significant differences were found in the knowledge scores for contact tracing and management of CAT 2 patients between public and private doctors (p-value < 0.05). We found that doctors trained by NTP had higher knowledge than their counterparts for the diagnosis and management of a new patient and for those with previous unsuccessful outcomes. These differences were statistically significant. However, differences in proportion of knowledge scores for counseling of the patient and contact screening were not significant between the two groups.

**Table 2:** Comparison of knowledge scores between public and private physicians in urban Pakistan

**Knowledge**	**Public**	**Private**	**p-value**
	**n = 100 (%)**	**n = 96 (%)**	
**Diagnosis of CAT 1 patients**			
Good	20 (20.0)	19 (20.0)	0.559
Fair	47 (47.0)	52 (54.0)	
Poor	33 (33.0)	25 (26.0)	
**Management of CAT 1 patients**			
Good	35 (35.0)	46 (48.0)	0.160
Fair	41 (41.0)	28 (29.0)	
Poor	24 (24.0)	22 (23.0)	
**Counseling of patients**			
Good	59 (59.0)	56 (58.0)	0.495
Poor	41 (41.0)	40 (42.0)	
**Contact screening**			
Good	54 (54.0)	41 (43.0)	0.044^*^
Poor	46 (46.0)	55 (57.0)	
**Diagnosis of CAT-2 patients**			
Good	18 (18.0)	10 (10.5)	0.298
Fair	30 (30.0)	35 (36.5)	
Poor	52 (52.0)	51 (53.0)	
**Management of CAT-2 patients**			
Good	38 (38.0)	20 (21.0)	0.028^*^
Fair	30 (30.0)	33 (34.0)	
Poor	32 (32.0)	43 (45.0)	

*p-value <0.05 was considered as significant.

Knowledge scores regarding diagnosis and management of TB patient: Good (≥75%), Fair (50.1-74.99%) and Poor (≤50).

Knowledge scores regarding counseling and contact screening of TB patient: Good (≥50%) and Poor (<50%).

CAT 1: Includes new cases of smear positive pulmonary tuberculosis or smear negative pulmonary tuberculosis with extensive parenchymal involvement or seriously ill extra pulmonary tuberculosis.

CAT 2: Includes previous treatment for more than four weeks in the past, and is found sputum smear positive pulmonary tuberculosis. This category includes: relapse, failure, treatment after default, and others (with smear positive).

**Table 3:** Comparison of knowledge scores between National TB Control Program (NTP) trained and untrained physicians in urban Pakistan

**Knowledge**	**NTP Training**	**No NTP Training**	**p-value**
	**n = 61 (%)**	**n = 135 (%)**	
**Diagnosis of CAT 1 patients**			
Good	22 (36.0)	18 (13.0)	<0.001^*^
Fair	29 (47.5)	69 (51.0)	
Poor	10 (16.5)	48 (36.0)	
**Management of CAT 1 patients**			
Good	33 (54.0)	47 (35.0)	0.007^*^
Fair	21 (34.5)	49 (36.0)	
Poor	7 (11.5)	39 (29.0)	
**Counseling of patients**			
Good	36 (59.0)	78 (58.0)	0.512
Poor	25 (41.0)	57 (42.0)	
**Contact screening**			
Good	30 (49.0)	65 (48.0)	0.506
Poor	31 (51.0)	70 (52.0)	
**Diagnosis of CAT-2 patients**			
Good	17 (28.0)	11 (8.0)	<0.001^*^
Fair	18 (29.5)	47 (35.0)	
Poor	26 (42.5)	77 (57.0)	
**Management of CAT-2 patients**			
Good	29 (47.5)	29 (21.5)	0.001^*^
Fair	17 (28.0)	46 (34.0)	
Poor	15 (24.5)	60 (44.5)	

*p-value <0.05 was considered as significant.

Knowledge scores regarding diagnosis and management of TB patient: Good (≥75%), Fair (50.1-74.99%) and Poor (≤50).

Knowledge scores regarding counseling and contact screening of TB patient: Good (≥50%) and Poor (<50%).

CAT 1: Includes new cases of smear positive pulmonary tuberculosis or smear negative pulmonary tuberculosis with extensive parenchymal involvement or seriously ill extra pulmonary tuberculosis.

CAT 2: Includes previous treatment for more than four weeks in the past, and is found sputum smear positive pulmonary tuberculosis. This category includes: relapse, failure, treatment after default, and others (with smear positive).

On logistic regression analysis, female sex, private employment status, and no NTP training were significantly associated with inadequate knowledge. However, treating ≤10 TB patients in the last one year was found to be significantly associated [OR 1.57; 95% CI: 1.258-1.996] with adequate knowledge scores. The final model showed that doctors being female, privately employed and not trained by NTP were associated with significantly inadequate knowledge of TB diagnosis and management as compared to the reference categories (Tables 4 and 5).

**Table 4:** Factors affecting the knowledge of physicians regarding tuberculosis management in urban Pakistan

**Variable**	**B (S.E)**	**OR (95% CI)**	**p-value**
**Age**			
20-35	1	1	
36-50	0.075 (0.38)	1.23 (0.635-2.379)	0.540
>50	0.072 (0.42)	1.15 (0.554-2.381)	0.709
**Sex**			
Male	1	1	
Female	0.078 (0.34)	1.23(0.650-0.957)	0.049^*^
**Years of practice**			
≤10	1	1	
>10	0.073 (0.52)	1.15 (0.652-2.035)	0.627
**Qualification**			
Diploma/fellow	1	1	
MBBS	0.093 (0.62)	1.5 (0.655-2.046)	0.614
**Employment status**			
Public	1	1	.
Private	0.071 (0.48)	1.15 (0.652-2.035)	0.627
**Number of patients treated per day**			
<50		1	
>50	−0.004 (0.29)	0.99 (0.564-1.789)	0.988
**Number of TB patients treated in last 1 year**			
>10	1	1	
≤10	0.090 (0.60)	1.57 (1.258-1.996)	0.0034^*^
**NTP training**			
Yes	1	1	
No	1.016(0.34)	2.72 (1.343-3.521)	<0.001^*^

*p-value < 0.05 was considered as significant.

**Table 5:** Multivariate logistic regression analysis of factors affecting the knowledge of physicians regarding tuberculosis management in urban Pakistan

**Variable**	**Adjusted B (S.E)**	**Adjusted OR (95% CI)**	**p-value**
**Sex**			
Male	1	1	
Female	1.015 (0.37)	2.76 (1.418-5.384)	0.003^*^
**Employment status**			
Public	1	1	
Private	0.591 (0.38)	1.50 (1.258-2.439)	0.034^*^
**Number of TB patients treated in last 1 year**			
≤10	1	1	
>10	0.232 (0.38)	1.16 (0.500-2.680)	0.732
**NTP training**			
Yes	1	1	
No	1.115 (0.38)	2.98 (1.286-3.225)	<0.001^*^

*p-value < 0.05 was considered as significant.

## Discussion

Knowledge of doctors regarding diagnosis and management of TB is imperative in eliminating TB through proper case management. Few surveys that have been conducted in Pakistan previously revealed that doctors working in the private sector have inadequate knowledge of TB [24]. Present study is the first attempt in Pakistan to identify the knowledge gaps between public and private doctors and factors affecting their knowledge in diagnosing and managing TB in a mega city of Pakistan.

Studies conducted in India and Oman reported significant differences in the knowledge of public and private doctors [25,26]. Findings of the present study showed that public doctors have more knowledge regarding management of CAT-2 patients as compared to private doctors (p-value 0.001). Higher proportion of patients with atypical tuberculosis symptoms (manifestation of extra pulmonary tuberculosis) presenting to the government hospitals due to lack of diagnosis by private care providers, availability of diagnostic facilities at a cheaper cost or possible referrals by private care providers could be a possible explanation for these observed differences.

Household contact screening of at risk individuals for TB is a relatively new concept in Pakistan and incorporated in NTP training of doctors since 2009. Findings of a systematic review and meta-analysis suggests that contact screening is an effective way to mend early case detection and decrease transmission especially in low and middle income high burden countries [27].

Although both the public and private doctors were scored less on knowledge items regarding contact screening, public doctors were found to have more knowledge regarding contact screening in our study. Like other previous surveys results [28], the regression analysis shows that private employment status was significantly associated with lesser knowledge score. Private sectors (formal/informal) are mainly unregulated in Pakistan and fewer opportunities are available for private doctors to update their knowledge. This, in addition to lack of motivation and non-availability of time required for training, are potential hindrances in practice of evidence based medicine. Although there is a recent acceleration in public private mix (PPM) activities by the NTP through involvement of NGOs and other stake holders, the need to train private health care providers on the standard guidelines of TB diagnosis and management still remains [29].

Our survey results indicate that public and private doctors used different methods to update their knowledge regarding management of TB patient; public doctors are more likely to update their knowledge to manage TB patients through academic meetings and books. On the other hand, Continuous Medical Education (CME) programs and formal NTP training of private doctors are the main sources for them to update their knowledge base. This is an important finding as CME platforms can be used to train private care providers on standard guidelines of diagnosis and management of TB in the future.

Amongst socio-demographic characteristics, female gender was found to be significantly associated with lower knowledge scores in univariate and multivariate analysis. Findings of a study conducted by Agboatwalla *et al.* in urban and rural areas of Sindh showed that overall female population has less knowledge about tuberculosis symptoms [30]. Physicians’ gender related differences in the knowledge, attitude and practices regarding TB have not been explored in any previous study in Pakistan and this finding is inconsistent with the results of a large survey conducted in Turkey where female doctors were found to have more knowledge scores as compared to their male counterparts [31]. Cultural factors, lack of interest of female doctors to update their knowledge or low exposure to TB patients, and particular carrier preferences for gynecology and surgery [32] are potential reasons for this finding in Pakistani context.

Results of this study have some important implications for policies and practices. From this survey, we found that only 31% of the doctors were trained by NTP on management of TB as per the standard guidelines. We did not find any significant differences in the number of doctors trained by NTP in public and private sector. However, those who were trained had more knowledge regarding diagnosis and management of CAT 1 and CAT 2 patients as compared to those who had not received any such training. On the other hand, we did not find any significant differences in the knowledge of contact screening and counseling between the two groups. Contact screening and counseling are important facets of TB prevention and treatment adherence [33]. Intensification of doctor’s knowledge on these aspects of TB care may prevent default and emergence of MDR TB.

In the present survey, knowledge of public and private doctors’ was assessed by using clinical scenario based vignettes. Vignettes or written case simulations have been used widely to assess clinical knowledge and competence of a group of providers [34]. It has been recognized by multiple studies that clinical vignettes or case simulation produced results that are comparable to the gold standard of the standardized patients and furthermore, this method of knowledge assessment is cost and time effective [34]. Face and construct validity of the clinical vignettes used in this study was conducted by the experts. In addition, findings of this study are generalizable as we have collected information from representative sample which comprised public (working in major public hospitals) and private doctors (from private hospitals as well as doctors in squatter settlement) of Karachi.

### Limitations of the study

Non response bias is a potential limitation of the study particularly with doctors working at independent clinics in squatter settlements because of their busy clinical schedule. We attempted to enhance the response rate by repeatedly contacting these doctors and making the questionnaire simple. Also, doctors who had more knowledge and experience of TB care were more likely to participate in the study and respond to the questionnaire which would result in overestimating physicians’ knowledge and experience. However this factor was controlled as a potential confounding factor in the multivariate regression analysis. We only recruited public and private doctors to compare their knowledge. However, no attempt has been made to compare the outcome of the tuberculosis patients treated by these doctors in order to verify their responses. There is a need to validate these findings of our study in Pakistan.

## Conclusion

The present study highlights the knowledge gap between the public and private doctors regarding the diagnosis and management of TB in a mega city of Pakistan. It is concluded that the private doctors have lesser knowledge regarding the diagnosis and management of TB as compared to public practitioners, particularly the management of CAT 2 patients. Training of the doctors (both public and private sectors) on the standard diagnosis and management guidelines of TB by NTP is vital in enhancing the knowledge of health care providers and is an important determinant of appropriate case management. In addition, strengthening of the currently implemented PPM model that involves all care providers, advocacy, communication and social mobilization is required in Pakistan to control and eliminate TB.

## Abbreviations

CME: Continuing medical education; DOTS: Directly observed treatment short course; MDG: Millennium Development Goal; MDR-TB: Multidrug-resistant tuberculosis; NTP: National tuberculosis control program; PPM: Public private mix; TB: Tuberculosis; UC: Union councils.

## Competing interests

The authors declare that they have no competing interest.

## Authors’ contributions

MN conceived the idea of the study and was involved in protocol writing, data collection, data analysis and manuscript writing. AK contributed in terms of data collection, data entry, data analysis and manuscript writing. AP participated in data analysis and manuscript writing. SA contributed in terms of data collection and manuscript writing. All authors approve the final version of the manuscript.

## Pre-publication history

The pre-publication history for this paper can be accessed here:

http://www.biomedcentral.com/1472-6963/13/439/prepub

## References

[B1] WHO WHO report 2010. Global TB control. WHO/HTM/TB/2010.7 2010 Geneva, Switzerland: WHO

[B2] World Health Organization WHO report 2011. Global TB Control. WHO/HTM/TB/2011.16 2011 Geneva, Switzerland: WHO

[B3] World Health Organization WHO report 2011. Global TB control: surveillance, planning, and financing 2009 Geneva, Switzerland: WHO

[B4] World Health Organization WHO progress report 2009. TB control in the Eastern Mediterranean Region Progress report 2009 Cairo, Egypt: WHO

[B5] Saleem T Khalid U Pakistan's battle with multi-drug resistant tuberculosis--establishing the ground rules J Pak Med Assoc 2012 62 80 22352115

[B6] World Health Organization WHO progress report 2011. Towards universal access to diagnosis and treatment of multidrug-resistant and extensively drug resistant TB by 2015 2011 Geneva, Switzerland: WHO

[B7] NTP Pakistan National TB control programme report-Performance indicators, National Data-Trends and analysis. National TB Control Programme Pakistan Available from: http://www.ntp.gov.pk/PerformanceIndicators.htm

[B8] NTP Pakistan Situation Analysis, Public-Private Partnership Models, Operational and Monitoring & Evaluation Guidelines for National TB Control Programme Pakistan. Project report 2006 2006 Available from: http://www.ntp.gov.pk/downloads/ppm/Finalreport_ppp_NTP08-09-06-PDF.pdf

[B9] Nishter S The gateway paper – health service delivery outside of the public sector in Pakistan J Pak Med Assoc 2006 56 S66 S77 17595834

[B10] Pakistan Medical Research Council National Health Survey of Pakistan 1990–94 1998 Islamabad: Ministry of Health

[B11] WHO The Global Plan to Stop TB 2006–2015: Action for Life Toward a World Free of Tuberculosis 2006 Geneva: WHO

[B12] WHO Stop TB Partnership and WHO Global Plan to Stop TB 2006–2015 2006 Geneva: World Health Organization report WHO/HTM/STB/2006.35

[B13] Raman AV Chadha VK Shashidhara AN Jaigopal MV Selvam A study of knowledge, attitude and practices of medical practitioners regarding TB and its control in a backward area of South India NTI Bulletin 2000 36 1–2 3 7

[B14] Datta K Bhatnagar T Murhekar M Private practitioners’ knowledge, attitude and practices about TB, Hooghly district, India Indian J Tuberc 2010 57 199 206 21141338

[B15] Shirzadi MR Majdzadeh R Pourmalek F Naraghi K Adherence of the private sector to national TB guidelines in the Islamic Republic of Iran, 2001–02 East Mediterr Health J 2003 9 796 804 15748076

[B16] Chen TC Lu PL Lin WR Lin CY Lin SH Lin CJ Lo WC Chen YH Diagnosis and treatment of pulmonary TB in hospitalized patients are affected by physician specialty and experience Am J Med Sci 2010 340 367 372 10.1097/MAJ.0b013e3181e92b06 20724908

[B17] Ahmed M Fatmi Z Ali S Ahmed J Ara N Knowledge, attitude and practice of private practitioners regarding TB-DOTS in a rural district of Sindh, Pakistan J Ayub Med Coll Abbottabad 2009 21 28 31 20364735

[B18] Shehzadi R Irfan M Zohra T Khan JA Hussain SF Knowledge regarding management of TB among general practitioners in northern areas of Pakistan J Pak Med Assoc 2005 55 174 176 15918634

[B19] United Nation World Urbanization Prospects: The 2009 Revision Population Database 2010 Online Database: United Nation population division

[B20] Pakistan Medical and Dental Association Practitioner statistics 2012 2012 Available from: http://www.pmdc.org.pk/Statistics/tabid/103/Default.aspx

[B21] Thaver IH Harpham T McPake B Garner P Private practitioners in the slums of Karachi: what quality of care do they offer? Soc Sci Med 1998 46 1441 1449 10.1016/S0277-9536(97)10134-4 9665574

[B22] Vandan N Ali M Prasad R Kuroiwa C Assessment of doctors’ knowledge regarding TB management in Lucknow, India: a public-private sector comparison Public Health 2009 123 484 489 10.1016/j.puhe.2009.05.004 19560176

[B23] Peabody JW Luck J Glassman P Dresselhaus TR Lee M Comparison of vignettes, standardized patients, and chart abstraction: a prospective validation study of 3 methods for measuring quality JAMA 2000 283 1715 1722 10.1001/jama.283.13.1715 10755498

[B24] Shah SK Sadiq H Khalil M Noor A Rasheed G Shah SM Ahmad N Do private doctors follow national guidelines for managing pulmonary TB in Pakistan? East Mediterr Health J 2003 9 776 788 15748074

[B25] Srivastava DK Mishra A Mishra S Chouksey M Jain P Gour N Bansal M A comparative assessment of KAP regarding TB and RNTCP among government and private practitioners in District Gwalior, India: an operational research Indian J Tuberc 2011 58 168 177 22533166

[B26] Al-Maniri AA Al-Rawas OA Al-Ajmi F De Costa A Eriksson B Diwan VK TB suspicion and knowledge among private and public general practitioners: questionnaire based study in Oman BMC Public Health 2008 8 177 10.1186/1471-2458-8-177 18501022 PMC2413224

[B27] De Muynck A Siddiqi S Ghaffar A Sadiq H TB control in Pakistan: critical analysis of its implementation J Pak Med Assoc 2001 51 41 47 11255999

[B28] Bell CA Duncan G Saini B **Knowledge, attitudes and practices of private sector providers of tuberculosis care: a scoping review** Int J Tuberc Lung Dis 2011 15 1005 17 10.5588/ijtld.10.0294 21669027

[B29] Naqvi SA Naseer M Kazi A Pethani A Naeem I Zainab S Fatmi Z Implementing a public-private mix model for TB treatment in urban Pakistan: lessons and experiences Int J Tuberc Lung Dis 2012 16 817 821 22507031 10.5588/ijtld.11.0440

[B30] Agboatwalla M Kazi GN Shah SK Tariq M Gender perspectives on knowledge and practices regarding tuberculosis in urban and rural areas in Pakistan East Mediterr Health J 2003 9 732 740 15748070

[B31] Dagli CE Cetin TA Hamit A Yilmaz P Gurdal Y Ekrem G Levent S Abdullah D Nurhan K A multicentre study of doctors’ approaches to the diagnosis and treatment of TB in Turkey J Infect Dev Ctries 2009 3 357 364 19759505 10.3855/jidc.243

[B32] Rehman A Rehman T Shaikh MA Yasmin H Asif A Kafil H Pakistani medical students’ specialty preference and the influencing factors J Pak Med Assoc 2011 61 713 718 22204259

[B33] Pankhurst LJ Anaraki S Lai KM Combining environmental assessment and contact investigations to make TB screening decisions Int J Tuberc Lung Dis 2012 16 1023 1029 10.5588/ijtld.11.0572 22691609

[B34] Miller GE The assessment of clinical skills/competence/performance Acad Med 1990 65 Suppl 9 S63 S67 2400509 10.1097/00001888-199009000-00045

